# Lower youth steps/day values observed at both high and low population density areas: a cross-sectional study in metropolitan Tokyo

**DOI:** 10.1186/s12889-018-6028-y

**Published:** 2018-09-20

**Authors:** Hiroki Sato, Shigeru Inoue, Noritoshi Fukushima, Hiroyuki Kikuchi, Tomoko Takamiya, Catrine Tudor-Locke, Yuki Hikihara, Shigeho Tanaka

**Affiliations:** 1 Department of Preventive Medicine and Public Health, Tokyo Medical University, 6-1-1 Shinjuku, Shinjuku-ku, Tokyo, 160-8402 Japan; 2 Oita Oka Hospital, 3-7-11 Nishitsurusaki, Oita, Oita, 870-0192 Japan; 3 Department of Kinesiology, School of Public Health and Health Sciences, University of Massachusetts Amherst, Amherst, MA 01002 USA; 4 Faculty of Creative Engineering, Chiba Institute of Technology, 2-1-1 Shibazono, Narashino, Chiba, 275-0023 Japan; 5 Department of Nutrition and Metabolism, National Institute of Health and Nutrition, National Institutes of Biomedical Innovation, Health and Nutrition, 1-23-1 Toyama, Shinjuku-ku, Tokyo, 162-8636 Japan

**Keywords:** Pedometer-determined step count, Population density, Cross-sectional study

## Abstract

**Background:**

Physical activity among children and adolescents (collectively, youth) is important to ensure adult health. Population density is a factor that affects physical activity via various environmental factors. However, the relationship between population density and physical activity among youth is not fully understood, especially in extremely high density area. The aim of this study was to examine the relationship between population density and physical activity of youth using pedometer-determined step data.

**Methods:**

A total of 13,688 youth between 6 to 15 years of age were identified from the 2011 Tokyo Metropolitan Survey of Physical Fitness, Physical Activity and Lifestyle. Participants were divided into five subgroups according to the population density of their municipality of residence. The population density’s fixed effects on in-school, out-of-school, and daily total step count adjusted for gender and school grade were estimated.

**Results:**

The lowest (< 2500 people/km^2^) and highest (> 10,000 people/km^2^) population density subgroups had significantly lower daily total step count and out-of-school step count than those of the reference population (5000–7500 people/km^2^). In contrast, in-school step count did not significantly differ according to population density.

**Conclusions:**

Both low population density and also high population density were related to lower step count. Low physical activity in high density areas has not been well documented in previous research. Considering population growth in urbanized area globally, these results suggest the importance of continued research of physical activity determinants in high population density areas.

**Electronic supplementary material:**

The online version of this article (10.1186/s12889-018-6028-y) contains supplementary material, which is available to authorized users.

## Background

The health benefits of physical activity and fitness in children and adolescents (collectively, youth) has been well documented [1]. Public health programs designed to increase physical activity among youth and promote healthy development may reduce the prevalence of risk factors for cardiovascular diseases, metabolic syndromes, or other diseases in adulthood. However, inactivity prevalence continued to be extremely high, with a global average of 78.4% for boys and 84.4% for girls in 2015 [2].

Physical activity in adolescents and adults is known to be influenced by environmental factors [3, 4]. Multiple studies have evaluated the relationship between environmental factors and youth physical activity, and several correlates have been identified [5–13]. Ding et al. reported that residential density (the number of dwellings divided by the land area) and land-use mix had the strongest influence on physical activity [10]. Some other studies reported that high residential or population densities (the number of people divided by the land area) have been significantly correlated with high physical activity levels among youth [6, 11, 13–16]. However, a study conducted among urban junior high school students in Nanjing, China reported a negative association between population density and physical activity [17]. They suggested that this inconsistency was a result of the high population density in Nanjing (2346 people/km^2^) compared to other previous studies (Atlanta in USA: 1220 people/km^2^, Adelaide in Australia: 1138 people/km^2^) [17]. Research conducted in London, England (5648 people/km^2^) showed that an increased population density was associated with a shorter walk home from school [5]. However, studies that have investigated youth physical activity levels in such high population density areas as Nanjing or London are few. In addition, most of these studies have relied upon self-reported estimates of physical activity which suffer from recall bias. Thus, the relationship between population density and physical activity among youth is still not completely understood, especially in extremely high population density areas.

The aim of this study was to investigate the uncertain relationship between population density and youth physical activity using pedometer-determined step data from the Tokyo Metropolitan Board of Education’s (TMBE) 2011 Tokyo Metropolitan Survey of Physical Fitness, Physical Activity and Lifestyle, which included youth participants between 6 and 15 years of age representing a range of very low and very high population density areas. Because the associations might differ depending upon where youth spent their time, relationships between population density and daily total step count, in-school step count, and out-of-school step count were assessed.

## Methods

### Data collection

Data were obtained from the Tokyo Metropolitan Survey of Physical Fitness, Physical Activity and Lifestyle 2011, which was a cross-sectional survey of pedometer-measured physical activity of youth. The survey was conducted by the TMBE and has been described elsewhere [18]. Step count data were collected from one class at every grade level of one public elementary school, junior high school, and high school in each Tokyo municipality during the 2011 fall term (September to November). The 62 Tokyo municipalities included 23 wards, 26 cities, five towns, and eight villages. The students participating in the original survey were between 6 to 18 years of age, and represented 62 public elementary schools, 62 public junior high schools, and 11 public high schools. Students of public elementary school and public junior high school live in the same municipality as where the school is located. The high school data were not included in this analysis because, unlike the lower grade levels, high school students may commute from several municipalities. Hence, this analysis is focused only on the elementary and junior high school students (i.e., 6 to 15 years of age). This analysis was designed to investigate the relationship between the population density of each municipality and the physical activity level of the children and adolescents living there.

In August 2011, each school held a survey orientation meeting for participants and their parents or guardians. The TMBE authority and the Tokyo Metropolitan Government approved the secondary use of these data for research purposes and all provided data were stripped of personal identifiers. This study was approved by the Medical Ethics Committee of the Tokyo Medical University. The study was conducted in accordance with the Declaration of Helsinki and the Ethical Guidelines for Medical and Health Research Involving Human Subjects provided by the Ministry of Health, Labour and Welfare, Japan.

### Step-determined physical activity

Participants were asked to wear a user-readable pedometer (EX-200; Yamasa Co., Ltd., Tokyo, Japan) during waking hours for 14 consecutive days. Yamasa is a Japanese corporation, known as Yamax in other countries, whose pedometers are commonly used in studies evaluating physical activity [19]. The EX-200 pedometer is currently available as the Yamax Power Walker EX-210. Participants could remove the device for water-based activities and while engaging in full-contact sports, such as judo. Following the original TMBE survey protocol, the number of steps per day during the first 7 days of monitoring was not recorded. The data for the remaining 7 days were evaluated. The participants recorded daily step counts on the study questionnaire. On weekdays, step counts were recorded three times: upon arrival at school, upon leaving school, and before going to bed. On holidays, steps were recorded only one time: before going to bed. Only weekday data were used in this study because one of the objectives was to investigate the relationship of in-school and out-of-school step counts with population density. In-school step counts were calculated by subtracting the step count upon arrival at school from the step count upon leaving school. Out-of-school step counts were obtained by subtracting the in-school step counts from the step count taken before going to bed.

### Demographic variables

Gender and school grade information were collected via a self-reported questionnaire.

### Data management

The pedometer step counts measured before going to bed that were below 1000 and above 30,000 were considered outliers and excluded from the analysis dataset, in accordance with Rowe’s procedure [20]. This procedure was used in the CANPLAY survey, which collected pedometer-based step count data of Canadian youth [21]. Because a single day of pedometer data can be used to accurately estimate physical activity levels for surveillance purposes, participants with at least one valid day of pedometer data were included in this analysis [21, 22]. If step count data were not reported for the time points defined as upon arrival at school, leaving school, or before going to bed, or if in-school and out-of-school step counts were below 0 (it would occur, for example, if more step counts were reported at the time point of leaving school than for that defined as before going to bed), the entire step count for the day was not entered. Step count data were also excluded if the participant’s gender or school grade were not reported. For each participant, the mean step count was calculated.

### Population density

Participants were classified by the population density of their residential municipalities. Schools to which they commuted were located in the same municipality of their residence. The 62 Tokyo municipalities were divided into five population density subgroups (lowest, lower, middle, higher, and highest) with densities of < 2500, 2500–5000, 5000–7500, 7500–10,000, and > 10,000 people/km^2^, respectively. Cut-off values were determined by reference to the past study in Nanjing which showed a negative relationship of youth physical activity and population density in higher density areas (> 12,665 people/km^2^) compared with lower density areas (< 3586 people/km^2^) [17]. Population density was obtained by dividing the number of residents by the habitable land area of the municipality. The habitable land area was calculated by subtracting the area of lakes and mountains from the total area. Population and habitable land area figures of each municipality were obtained from the regional statistics report of the prefectures and municipalities by the Japan Ministry of Internal Affairs and Communications [23].

### Statistical analysis

Data were reported as means with a standard deviation for continuous variables or as frequency and percentage for categorical variables. Percentage of participants whose step count exceeded the recommended step count was presented for each population density subgroup. Cut-off values were obtained from the results of the paper written by Tudor-Locke et al., although the proposed values were preliminary because they were based on a limited number of relevant studies [24]. Cut-off values are as follows; boys aged 6–12 years: 13,000 steps, girls aged 6–12 years: 11000 steps, adolescents aged 12–19 years: 10,000 steps. Because step patterns are different by gender and age [24], analyses were conducted as follows. The relationships between population density subgroups and daily total, in-school, and out-of-school step counts were evaluated by gender using a linear mixed model with the municipality as a random effect. The school grade of each participant was included into the model as fixed effect.

In the subgroup analysis, population-density-related differences in step counts were compared in the gender and school grade groups. Participants were divided into six groups by gender and school grade: low elementary school (first to third grade), high elementary school (fourth to sixth grade), and junior high school (first to third grade). Each model was adjusted for school grade. The ages of these grade levels corresponded to 6 to 9 years, 9 to 12 years, and 12 to 15 years of age, respectively. A *p*-value < 0.05 was considered statistically significant. All statistical analyses were performed using SAS University Edition (SAS Institute Inc., Cary, NC, USA).

## Results

### Participant characteristics

**Fig. 1:**
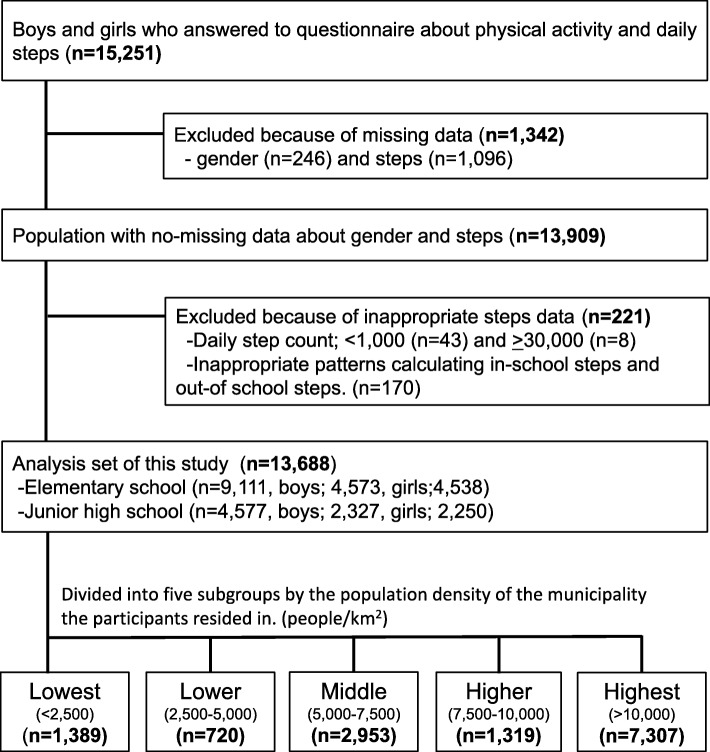
Flow diagram of participant selection

Of the 15,251 questionnaire respondents who also provided daily step count data, 1342 were excluded because of missing gender and/or step count data, and 221 were excluded because of insufficient step data (Fig. 1). The remaining 13,688 participants (6900 boys and 6788 girls) were included in this study and allocated to the five population density subgroups. Table 1 shows the details of municipalities divided by population density; Table 2 lists the number of participants by gender and school grade within each population density subgroup.

**Table 1:** Municipality characteristics by population density subgroup

	Total		Population density									
			Lowest		Lower		Middle		Higher		Highest	
No. of municipalities	62		13		3		12		5		29	
Type of municipality^a^												
Wards	23	(37.1)	0	(0.0)	1	(33.3)	0	(0.0)	0	(0.0)	22	(75.9)
City	26	(41.9)	0	(0.0)	2	(66.7)	12	(100.0)	5	(100.0)	7	(24.1)
Town	5	(8.1)	5	(8.1)	0	(0.0)	0	(0.0)	0	(0.0)	0	(0.0)
Village	8	(12.9)	8	(12.9)	0	(0.0)	0	(0.0)	0	(0.0)	0	(0.0)
No. of people^b,c^	212.7	(213.6)	6.6	(9.2)	89.3	(46.3)	171.6	(164.8)	157.6	(68.9)	344.3	(217.6)
Area (km^2^)^c^												
Total area	35.1	(40.7)	59.8	(61.8)	62.7	(46.7)	35.5	(50.4)	17.6	(8.0)	24.2	(16.2)
Residential area	22.4	(18.2)	15.7	(16.1)	26.2	(13.3)	26.5	(27.2)	17.4	(8.0)	24.1	(16.1)
Population density^c,d^	9176	(6228)	532	(735)	3524	(675)	6555	(810)	9127	(244)	14,728	(3315)

^a^Data were presented as frequency (percentage)

^b^The number of people was shown as (× 1000)

^c^Data were presented as mean (standard deviation)

^d^Population density was calculated by dividing the number of people by residential area for each municipality

**Table 2:** The number of participants by gender and school grades in each population density subgroup

	Total	Population density									
		Lowest		Lower		Middle		Higher		Highest	
Boys											
Elementary school											
Low grade	2218	227	(10.2)	90	(4.1)	440	(19.8)	219	(9.9)	1242	(56.0)
1st, 6–7 y.o.^a^	760	76	(10.0)	29	(3.8)	160	(21.1)	79	(10.4)	416	(54.7)
2nd, 7–8 y.o.	737	73	(9.9)	32	(4.3)	145	(19.7)	74	(10.0)	413	(56.0)
3rd, 8–9 y.o.	721	78	(10.8)	29	(4.0)	135	(18.7)	66	(9.2)	413	(57.3)
High grade	2355	232	(9.9)	146	(6.2)	517	(22.0)	231	(9.8)	1229	(52.2)
4th, 9–10 y.o.	788	80	(10.2)	45	(5.7)	162	(20.6)	83	(10.5)	418	(53.0)
5th, 10–11 y.o.	756	71	(9.4)	43	(5.7)	174	(23.0)	76	(10.1)	392	(51.9)
6th, 11–12 y.o.	811	81	(10.0)	58	(7.2)	181	(22.3)	72	(8.9)	419	(51.7)
Junior high school											
All grade	2327	260	(11.2)	120	(5.2)	467	(20.1)	234	(10.1)	1246	(53.5)
1st, 12–13 y.o.	820	79	(9.6)	40	(4.9)	171	(20.9)	74	(9.0)	456	(55.6)
2nd, 13–14 y.o.	749	96	(12.8)	33	(4.4)	166	(22.2)	71	(9.5)	383	(51.1)
3rd, 14–15 y.o.	758	85	(11.2)	47	(6.2)	130	(17.2)	89	(11.7)	407	(53.7)
Total	6900	719	(10.4)	356	(5.2)	1424	(20.6)	684	(9.9)	3717	(53.9)
Girls											
Elementary school											
Low grade	2253	208	(9.2)	109	(4.8)	481	(21.3)	210	(9.3)	1245	(55.3)
1st, 6–7 y.o.	733	55	(7.5)	32	(4.4)	156	(21.3)	68	(9.3)	422	(57.6)
2nd, 7–8 y.o.	756	59	(7.8)	36	(4.8)	169	(22.4)	75	(9.9)	417	(55.2)
3rd, 8–9 y.o.	764	94	(12.3)	41	(5.4)	156	(20.4)	67	(8.8)	406	(53.1)
High grade	2285	215	(9.4)	130	(5.7)	526	(23.0)	214	(9.4)	1200	(52.5)
4th, 9–10 y.o.	746	79	(10.6)	33	(4.4)	165	(22.1)	75	(10.1)	394	(52.8)
5th, 10–11 y.o.	771	64	(8.3)	42	(5.4)	177	(23.0)	69	(8.9)	419	(54.3)
6th, 11–12 y.o.	768	72	(9.4)	55	(7.2)	184	(24.0)	70	(9.1)	387	(50.4)
Junior high school											
All grade	2250	247	(11.0)	125	(5.6)	522	(23.2)	211	(9.4)	1145	(50.9)
1st, 12–13 y.o.	782	88	(11.3)	38	(4.9)	184	(23.5)	68	(8.7)	404	(51.7)
2nd, 13–14 y.o.	703	82	(11.7)	42	(6.0)	163	(23.2)	64	(9.1)	352	(50.1)
3rd, 14–15 y.o.	765	77	(10.1)	45	(5.9)	175	(22.9)	79	(10.3)	389	(50.8)
Total	6788	670	(9.9)	364	(5.4)	1529	(22.5)	635	(9.4)	3590	(52.9)

Data were presented as frequency (percentage)

^a^y.o.; years old

### Step count by population density subgroups

**Fig. 2:**
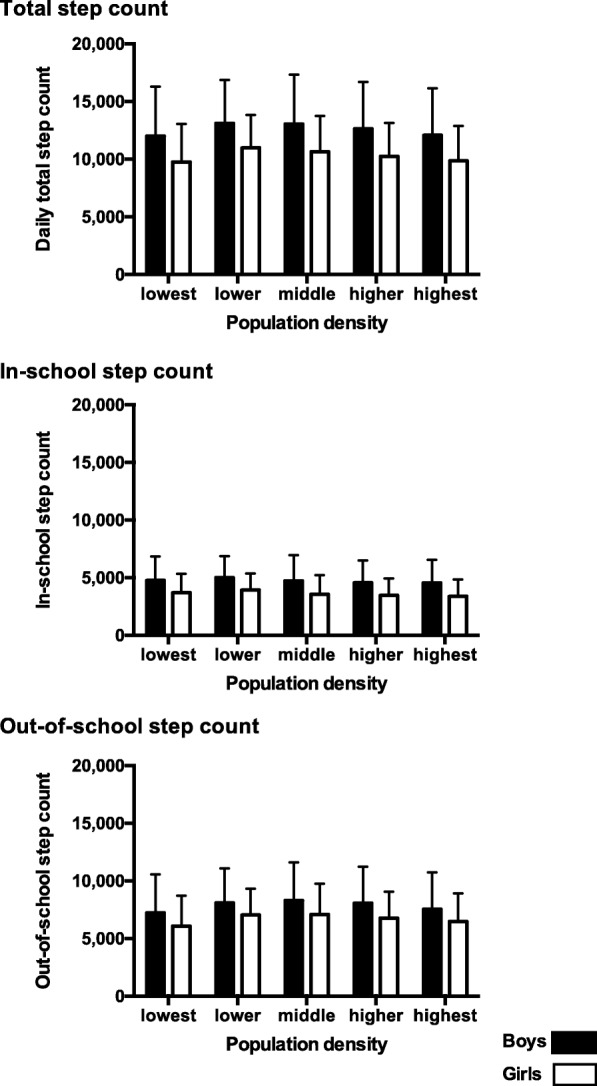
Step count by population density subgroups for all boys and girls. Step counts were shown as means with bars of standard deviations

The number of weekdays with valid step count data for each participant was summarized in Table 3. More than 80% of participants had valid step count data with > 3 days. The daily total step count, which included both in-school and out-of-school step counts, in each population density subgroup is shown by gender (Fig. 2 and Additional file 1: Table S1). For both boys and girls, the daily total step count data displayed an inverted U-shaped distribution with a peak in the lower density subgroup and a nadir in the lowest subgroup. The highest daily total step count was observed among the lower population density subgroup (boys: 13,105 steps; girls: 10,975 steps). The lowest daily total step count was observed in the lowest population density subgroup (boys: 11,993 steps; girls: 9751 steps).

**Table 3:** The number of weekdays with valid step count data by gender and school grades

	Number of weekdays with valid step count data^a^									
	1		2		3		4		5	
Total boys and girls	923	(6.7)	1408	(10.3)	2607	(19.0)	4242	(31.0)	4508	(32.9)
Boys										
Total boys	507	(7.3)	753	(10.9)	1292	(18.7)	2127	(30.8)	2221	(32.2)
Low grade, elementary school	168	(7.6)	299	(13.5)	430	(19.4)	666	(30.0)	655	(29.5)
High grade, elementary school	171	(7.3)	214	(9.1)	386	(16.4)	709	(30.1)	875	(37.2)
Junior high school	168	(7.2)	240	(10.3)	476	(20.5)	752	(32.3)	691	(29.7)
Girls										
Total girls	416	(6.1)	655	(9.6)	1315	(19.4)	2115	(31.2)	2287	(33.7)
Low grade, elementary school	146	(6.5)	252	(11.2)	439	(19.5)	686	(30.4)	730	(32.4)
High grade, elementary school	129	(5.6)	188	(8.2)	371	(16.2)	655	(28.7)	942	(41.2)
Junior high school	141	(6.3)	215	(9.6)	505	(22.4)	774	(34.4)	615	(27.3)

^a^Data were presented as frequency (percentage)

The in-school step count by population density subgroup was similar to that of the daily total step count; however, a nadir was observed in the highest subgroup for both genders. The out-of-school step count also had an inverted U-shaped distribution with a peak in the middle population density subgroup and a nadir in the lowest subgroup.

**Fig. 3:**
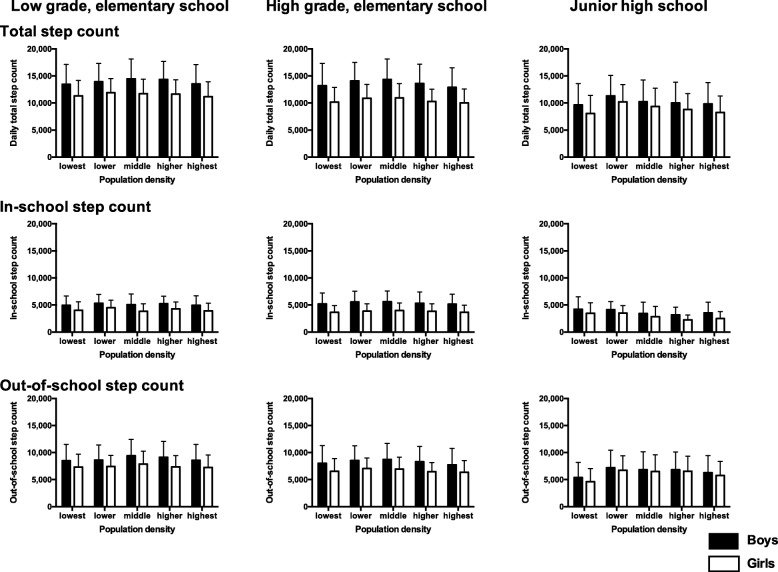
Step count by population density subgroups by gender and school grades. Step counts were shown as means with bars of standard deviations

**Fig. 4:**
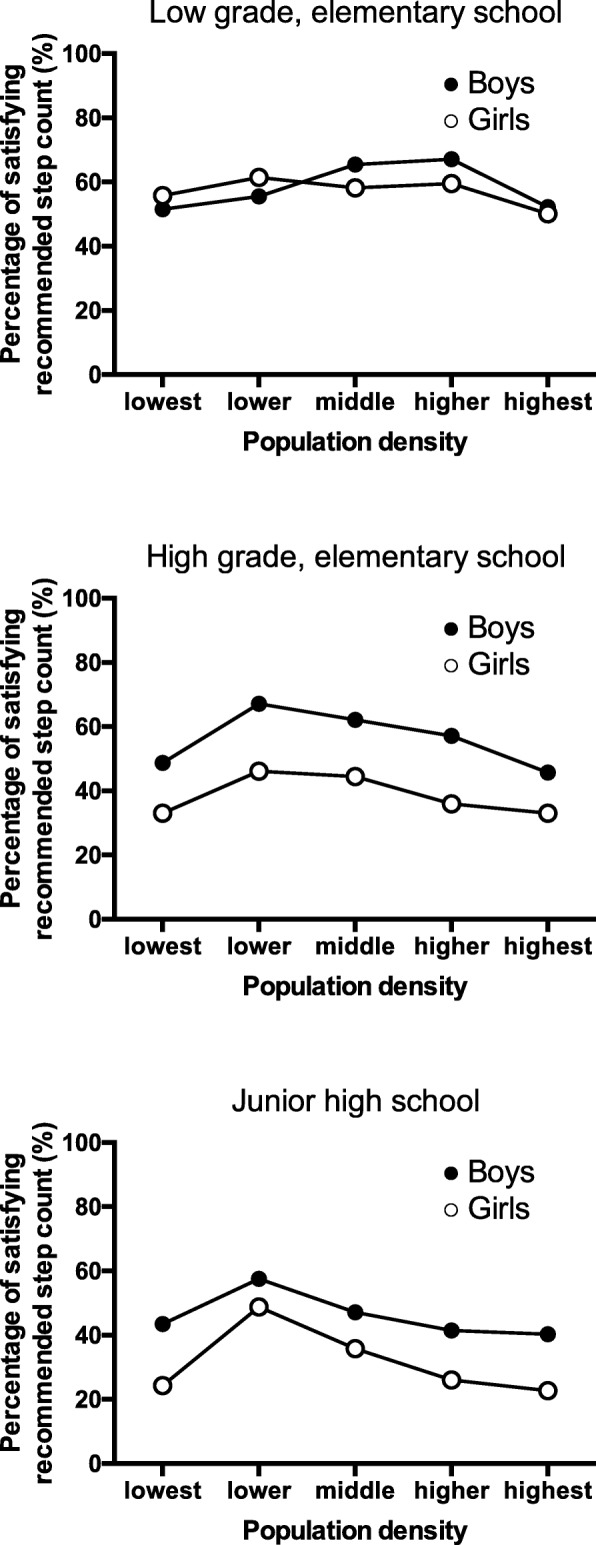
Percentage of participants with recommended daily total step count by population density subgroup

The daily total step count of each population density subgroup by school grade was similar for both genders (Fig. 3 and Additional file 1: Table S1). An inverted U-shaped distribution was shown in every gender and school grade stratified group. Percentage of participants with adequate daily total step count by gender and age group were presented in Fig. 4 and Additional file 2: Table S2. Inverted U-shaped distributions displaying a peak in the lower density subgroup were shown among participants except boys and girls in the low grade of elementary school. Percentage of participants with adequate step count was low in girls and gradually decreased by age, compared within the same population density subgroup. The in-school step count of each population density subgroup differed by gender and school grade. With the exclusion of higher grade (i.e., relatively older) elementary school students, the in-school step count varied between population density subgroups. The out-of-school step count displayed an inverted U-shaped distribution with a peak in the lower or the middle population density subgroup among most (5/6) of the gender and school grade groupings.

### Fixed effects of population density

The fixed effects of population density on daily total, in-school, and out-of-school step count by gender were estimated using a linear mixed model (Table 4). Participants living in both the lowest and highest population density subgroups had significantly lower estimates, as compared with the reference middle subgroup. For boys, the lowest and highest population density subgroups had significantly lower total step counts than that of the reference subgroup, differing by 1077 steps (standard error [SE], 458, *p* = 0.019) and 987 steps (SE, 373, *p* = 0.008), respectively. A similar trend was observed among girls and for out-of-school step count in both genders. Yet, the in-school step count did not significantly differ between population density subgroups.

**Table 4:** Fixed effect estimates of population density and school grade on step counts for all boys and girls

	Lowest		Lower		Middle		Higher		Highest		School grade	
	Estimates (SE)^a^		Estimates (SE)		Estimates (SE)		Estimates (SE)		Estimates (SE)		Estimates (SE)	
Daily total step count												
Boys	−1077	(458)	302	(701)	ref.	–	−371	(576)	−987	(373)	−617	(17)
	*p* = 0.02		*p* = 0.67		–		*p* = 0.52		*p* = 0.01		*p* < 0.01	
Girls	−1059	(414)	441	(636)	ref.	–	−409	(524)	−868	(338)	−457	(13)
	*p* = 0.01		*p* = 0.49		–		*p* = 0.44		*p* = 0.01		*p* < 0.01	
In-school step count												
Boys	119	(238)	385	(365)	ref.	–	−153	(300)	−180	(194)	−222	(9)
	*p* = 0.62		*p* = 0.29		–		*p* = 0.61		*p* = 0.35		*p* < 0.01	
Girls	233	(231)	424	(358)	ref.	–	−110	(295)	−207	(190)	−200	(7)
	*p* = 0.31		*p* = 0.24		–		*p* = 0.71		*p* = 0.28		*p* < 0.01	
Out-of-school step count												
Boys	−1192	(339)	−83	(515)	ref.	–	−219	(422)	−806	(274)	−395	(14)
	*p* < 0.01		*p* = 0.87		–		*p* = 0.60		*p* < 0.01		*p* < 0.01	
Girls	−1259	(298)	18	(453)	ref.	–	−299	(373)	−660	(241)	−257	(11)
	*p* < 0.01		*p* = 0.97		–		*p* = 0.42		*p* = 0.01		*p* < 0.01	

^a^Estimates of fixed effect were shown with standard errors

Results of the population density subgroup analysis by gender and school grade are presented in Table 5. An inverted U-shaped distribution similar to that of total step count by population density subgroup apparent for all boys and all girls was only observed in junior high school girls. When out-of-school and in-school step counts were considered separately, the out-of-school step count trend indicated an inverted U-shaped distribution in most population density subgroups. Conversely, the in-school step count of junior high school students in the lowest population density subgroup was significantly higher than that of the reference group.

**Table 5:** Fixed effect estimates of population density and school grade on step counts by gender and school grade

	Lowest		Lower		Middle		Higher		Highest		School grade	
	Estimates (SE)^a^		Estimates (SE)		Estimates (SE)		Estimates (SE)		Estimates (SE)		Estimates (SE)	
Daily total step count												
Boys												
Low grade, elementary school	− 1072	(582)	− 577	(871)	ref.	–	4	(700)	− 914	(455)	129	(89)
	*p* = 0.07		*p* = 0.51		–		*p* = 1.00		*p* = 0.04		*p* = 0.15	
High grade, elementary school	− 1274	(665)	−113	(993)	ref.	–	− 696	(820)	− 1390	(531)	−287	(88)
	*p* = 0.06		*p* = 0.91		–		*p* = 0.40		p = 0.01		p < 0.01	
Junior high school	− 569	(658)	1094	(1000)	ref.	–	− 130	(818)	− 418	(532)	− 918	(94)
	*p* = 0.39		*p* = 0.27		–		p = 0.87		*p* = 0.43		p < 0.01	
Girls												
Low grade, elementary school	− 686	(505)	209	(757)	ref.	–	−63	(620)	− 545	(400)	−50	(68)
	*p* = 0.17		*p* = 0.78		–		*p* = 0.92		p = 0.17		*p* = 0.46	
High grade, elementary school	−993	(508)	54	(755)	ref.	–	− 628	(623)	− 916	(402)	− 416	(62)
	*p* = 0.05		*p* = 0.94		–		p = 0.31		p = 0.02		p < 0.01	
Junior high school	− 1230	(577)	905	(867)	ref.	–	− 483	(715)	−1100	(462)	− 778	(75)
	*p* = 0.03		*p* = 0.30		–		*p* = 0.50		p = 0.02		p < 0.01	
In-school step count												
Boys												
Low grade, elementary school	101	(336)	260	(510)	ref.	–	187	(414)	−43	(268)	153	(43)
	*p* = 0.76		p = 0.61		–		*p* = 0.65		p = 0.87		p < 0.01	
High grade, elementary school	− 439	(350)	34	(523)	ref.	–	− 273	(432)	−455	(280)	−59	(46)
	*p* = 0.21		*p* = 0.95		–		*p* = 0.53		*p* = 0.10		*p* = 0.20	
Junior high school	788	(349)	693	(533)	ref.	–	− 260	(437)	95	(284)	− 183	(48)
	p = 0.02		*p* = 0.19		–		*p* = 0.55		*p* = 0.74		p < 0.01	
Girls												
Low grade, elementary school	368	(330)	628	(509)	ref.	–	452	(418)	67	(270)	109	(33)
	p = 0.27		*p* = 0.22		–		p = 0.28		*p* = 0.80		< 0.01	
High grade, elementary school	− 341	(280)	−54	(419)	ref.	–	− 144	(345)	− 290	(223)	− 129	(32)
	p = 0.22		*p* = 0.90		–		*p* = 0.68		p = 0.19		< 0.01	
Junior high school	822	(360)	678	(554)	ref.	–	− 571	(456)	−324	(295)	− 155	(34)
	p = 0.02		p = 0.22		–		p = 0.21		p = 0.27		< 0.01	
Out-of-school step count												
Boys												
Low grade, elementary school	− 1134	(519)	− 837	(782)	ref.	–	−177	(632)	− 869	(410)	−25	(73)
	p = 0.03		p = 0.28		–		p = 0.78		p = 0.03		*p* = 0.73	
High grade, elementary school	− 815	(456)	− 149	(662)	ref.	–	−424	(547)	− 938	(355)	− 227	(73)
	p = 0.07		*p* = 0.82		–		p = 0.44		p = 0.01		p < 0.01	
Junior high school	− 1358	(471)	403	(707)	ref.	–	128	(578)	− 513	(376)	− 736	(77)
	p < 0.01		*p* = 0.57		–		*p* = 0.83		p = 0.17		p < 0.01	
Girls												
Low grade, elementary school	− 973	(384)	− 421	(569)	ref.	–	− 517	(466)	− 613	(301)	−162	(57)
	p = 0.01		p = 0.46		–		p = 0.27		p = 0.04		p < 0.01	
High grade, elementary school	−627	(368)	110	(539)	ref.	–	−483	(445)	− 625	(287)	− 288	(52)
	*p* = 0.09		*p* = 0.84		–		p = 0.28		p = 0.03		p < 0.01	
Junior high school	−2016	(413)	235	(607)	ref.	–	95	(501)	−762	(324)	− 626	(66)
	p < 0.01		*p* = 0.70		–		*p* = 0.85		p = 0.02		p < 0.01	

^a^ Estimates of fixed effect were shown with standard errors

## Discussion

This study investigated the relationship between population density and daily total and segmented (by in- and out-of-school conditions) step counts among 6- to 15-year-olds living in Tokyo. Daily total step counts for the five population density subgroups exhibited a distribution with an inverted U-shape. The distribution of the percentage of participants with adequate daily total step count by population density subgroups was similar to that of the daily total step count except for participants enrolled in low grade of elementary school. Both high and low population densities were associated with lower physical activity levels relative to the reference group, and this trend was specifically explained by differences in out-of-school physical activity levels. Previous studies have reported positive correlations between youth physical activity and population density [6, 11, 13–16], while some studies have indicated negative associations [5, 17]. As previously mentioned, these discrepant findings may have resulted from the spectrum of population densities organic to the study setting. The mean population density of Tokyo (9176 people/km^2^) is much higher than that studied in previous research [23]. Tokyo itself consists of various types of municipalities (i.e. islands, mountainous regions, small cities, and a highly urbanized city) ranging from 53 to 22,308 people/km^2^ [23]. To the best of our knowledge, this large and broadly inclusive survey is the first study to report both positive and negative associations correlations, showing low physical activity levels in both low and extremely high population densities.

Mixed model analysis suggested that daily total step count of youths living in the lowest subgroup and those in the highest subgroup were about 1000 step count and 500 step counts less than those in the middle population density subgroup, respectively. These step count differences are about 5–10% of the recommended step count. However, a public health-oriented strategy aimed at increasing the physical activity distribution by even this relatively small incremental difference would theoretically produce a great benefit for whole population [25]. As shown in Additional file 2: Table S2, an approximate 500–1000 step difference in daily physical activity was associated with an approximate 5–15% increased probability that participants would achieve recommended daily step count values. For example, the probability of participants achieving the recommended daily step count among boys in high grade of elementary school living in the middle population density subgroup was 62.1%, compared with that among those who living in the highest subgroup (45.7%).

Our study analyzed previously surveyed in-school and out-of-school step count. This was helpful for assessing whether the influence of population density on total daily step counts was based on in-school or out-of-school step counts. The relationship between population density and out-of-school step count was similar to that observed for the total daily step count. In contrast, the in-school step count did not significantly differ between population density subgroups suggesting that a more standardized educational experience provides a more uniform effect on physical activity within school hours. Differences in total daily step count between population density subgroups were exclusively attributed to apparent differences in out-of-school step counts. This implies that environmental contributions related to population density had more of an influence on out-of-school step counts and less of an influence on in-school physical activity.

This study was not able to identify the specific population density-related environmental factors that influenced youth physical activity because the original data set used did not contain information on neighborhood environments. But we estimate that differences in step count between population density subgroups might be due to differences in commuting modes influenced by distance to school and the levels of automobile traffic or crime, which also relate to population density. Although higher population density is generally associated with more walkable neighborhood environments, consisting of high density, high land use mix, and high street connectivity, there have been some studies suggesting unfavorable effects of higher population density on youth physical activity [5, 26–30]. For example, Larsen et al. investigated transportation modes between home and school among students 11 to 13 years of age living in London, England, and reported that higher residential densities were associated with a lower travel-related physical activity level [5]. Oliver et al. reported that high traffic speeds around schools were associated with low out-of-school physical activity on weekdays among students 9 to 13 years of age living in Auckland, New Zealand [26]. Some studies have also reported that increased levels of automobile traffic and crime related to increased residential density prevent youth from walking [5, 27, 28]. D’Haese et al. and Kurka et al. reported that parents living in a community with high population density tended to be unsupportive of youth walking to school or engaging in out-of-school physical activities [29, 30]. In contrast, as already mentioned above, many other studies indicated positive relationships between youth physical activity levels and residential/population density [6, 10, 11, 13–16]. In this study, we categorized subgroups by population densities of Tokyo metropolitan, and the density of the “lower” subgroup was 2500–5000 people/km^2^, which means that the “lower” subgroup in this study was not necessary low population density compared with previous studies [6, 11, 13–16]. Most of target cities in past studies would be included in the “lower” or “lowest” subgroup in this study [6, 11, 13–16], suggesting that the “lower” subgroup herein may relatively correspond to high population density in previous studies, which was reported to be favorable for physical activity. Additionally, we think that the observed difference in step count between population density might be caused, as mentioned above, by commuting modes influenced by distance to school and the levels of automobile traffic which relating to population density. Youths living in municipalities categorized into the lowest subgroup would likely commute to school by parents’ automobile rather than by walking because of living a further distance from their school. On the other hand, youths living in the highest subgroup may walk less to school because of increased levels of automobile traffic and crime related to increased residential density.

This study has several strengths. First, the population density of central Tokyo is much higher than those of previous studies that have investigated youth physical activity and population density. Furthermore, metropolitan Tokyo consists of various types of municipalities ranging widely in population density from 53 to 22,008 people/km^2^ within a single prefecture. This broad range facilitated analysis that clearly showed low physical activity among youth in low population density areas, but also in extremely high population density areas. Second, the study employed objective assessment methods, that is, pedometer-determined physical activity from a large sample size of 13,688 participants and population density data was based on governmental statistics. The number of participants in this study is large compared with previous studies with this similar research question [6, 11, 13–17]. Since previous studies have reported a positive or negative relationship between youth physical activity and population density, this study addressed this inconsistency by recruiting a large population size from different municipalities within a very wide range of population densities.

This study has several limitations. First, population density was calculated from the total number of people and the habitable land area of each municipality obtained from the regional statistics report of the prefectures and municipalities by the Japan Ministry of Internal Affairs and Communications. Thus, the population density used in this study may not accurately reflect the local population density of each participant’s neighborhood. Second, the relationship between step count and population density was investigated with considerations for only gender and school grade because the TMBE survey did not originally collect information about neighborhood environmental factors. In addition, some socioeconomic characteristics related to population density may have influenced the results. Despite these limitations, the findings of this study are important because this is the first report to show that both a low population density and a high population density are related to low levels of objectively monitored physical activity. Low physical activity in high population density area has not been well documented and recognized. As a consequence, determinants of physical activity in such areas have been understudied. Continued population growth is anticipated in urbanized areas globally. These results suggest the importance of studies of physical activity determinants in extremely high density areas in an effort to inform effective interventions.

## Conclusions

In conclusion, low population density but also high population density were both related to low physical activity levels in youth between 6 and 15 years of age living in Tokyo. Population density-related differences in daily total step count were related to differences in out-of-school step counts rather than in-school step counts.

## Additional files

Additional file 1: **Table S1.** Daily total, in-school and out-of-school step count by population density subgroup. (DOCX 21 kb)

Additional file 2: **Table S2.** Frequency and percentage of participants with adequate daily total step count by population density subgroups. (DOCX 15 kb)

## Abbreviations

SE: Standard error
TMBE: Tokyo Metropolitan Board of Education
