# Supplementary material for: Lower youth steps/day values observed at both high and low population density areas: a cross-sectional study in metropolitan Tokyo
Source: BMC Public Health. 2018 Sep 20;18:1132. doi: 10.1186/s12889-018-6028-y (PMC6149053; doi:10.1186/s12889-018-6028-y)
Supplement: Supplementary file 1 — Table S1. Daily total, in-school and out-of-school step count by population density subgroup. (DOCX 21 kb) [file 12889_2018_6028_MOESM1_ESM.docx]

| Table S1. Daily total, in-school and out-of-school step count by population density subgroup. | | | | | | | | | | | | | | | | | | | | | | | |  |  |
| --- | --- | --- | --- | --- | --- | --- | --- | --- | --- | --- | --- | --- | --- | --- | --- | --- | --- | --- | --- | --- | --- | --- | --- | --- | --- |
|  |  |  |  |  | |  |  | Population density | | | | | | | | | | | | | | | | |  |
|  |  |  |  | Total | | |  | Lowest | |  | Lower | |  | Middle | | |  | Higher | | |  | Highest | | | |
| **Boys** | | |  |  | |  |  |  |  |  |  |  |  |  | |  |  |  | |  |  |  |  | | |
| Total boys | | | | | | |  |  | |  |  | |  |  | | |  |  | | |  |  | | | |
|  | Total step count | |  | 12377 | | (4142) |  | 11993 | (4304) |  | 13105 | (3777) |  | 13041 | (4285) | |  | 12621 | (4071) | |  | 12081 | (4059) | | |
|  |  | In-school |  | 4630 | | (2050) |  | 4766 | (2080) |  | 5011 | (1863) |  | 4726 | (2215) | |  | 4555 | (1937) | |  | 4545 | (2009) | | |
|  |  | Out-of-school | | | 7746 | (3234) |  | 7227 | (3333) |  | 8094 | (3001) |  | 8315 | (3285) | |  | 8066 | (3173) | |  | 7537 | (3191) | | |
| Low grade, elementary school | | | | | | |  |  |  |  |  |  |  |  |  | |  |  |  | |  |  |  | | |
|  | Total step count | |  | 13811 | | (3582) |  | 13454 | (3692) |  | 13918 | (3400) |  | 14469 | (3663) | |  | 14359 | (3341) | |  | 13539 | (3550) | | |
|  |  | In-school |  | 5017 | | (1766) |  | 4949 | (1717) |  | 5310 | (1672) |  | 5067 | (1972) | |  | 5225 | (1395) | |  | 4954 | (1761) | | |
|  |  | Out-of-school | | | 8794 | (2979) |  | 8505 | (3024) |  | 8608 | (2785) |  | 9402 | (3035) | |  | 9134 | (2932) | |  | 8586 | (2941) | | |
| High grade, elementary school | | | | | | |  |  | |  |  | |  |  | | |  |  | | |  |  | | | |
|  | Total step count | |  | 13389 | | (3743) |  | 13205 | (4112) |  | 14074 | (3451) |  | 14336 | (3786) | |  | 13629 | (3571) | |  | 12899 | (3629) | | |
|  |  | In-school |  | 5299 | | (1938) |  | 5184 | (2025) |  | 5552 | (1994) |  | 5613 | (1966) | |  | 5321 | (2064) | |  | 5155 | (1861) | | |
|  |  | Out-of-school | | | 8090 | (3031) |  | 8021 | (3291) |  | 8522 | (2738) |  | 8723 | (2989) | |  | 8308 | (2840) | |  | 7744 | (3017) | | |
| Junior high school | | |  |  | |  |  |  |  |  |  |  |  |  |  | |  |  |  | |  |  |  | | |
|  | Total step count | |  | 9985 | | (3967) |  | 9635 | (3949) |  | 11316 | (3811) |  | 10263 | (4006) | |  | 10001 | (3850) | |  | 9822 | (3967) | | |
|  |  | In-school |  | 3585 | | (2000) |  | 4233 | (2300) |  | 4129 | (1485) |  | 3423 | (2087) | |  | 3173 | (1430) | |  | 3535 | (1996) | | |
|  |  | Out-of-school | | | 6400 | (3207) |  | 5403 | (2796) |  | 7187 | (3267) |  | 6840 | (3301) | |  | 6828 | (3292) | |  | 6287 | (3175) | | |
| **Girls** | | |  |  | |  |  |  |  |  |  |  |  |  |  | |  |  |  | |  |  |  | | |
| Total girls | | | | | | |  |  | |  |  | |  |  | | |  |  | | |  |  | | | |
|  | Total step count | |  | 10115 | | (3084) |  | 9751 | (3298) |  | 10975 | (2872) |  | 10637 | (3103) | |  | 10243 | (2874) | |  | 9851 | (3046) | | |
|  |  | In-school |  | 3480 | | (1545) |  | 3684 | (1654) |  | 3928 | (1425) |  | 3556 | (1650) | |  | 3460 | (1490) | |  | 3367 | (1485) | | |
|  |  | Out-of-school | | | 6635 | (2501) |  | 6067 | (2662) |  | 7048 | (2273) |  | 7081 | (2659) | |  | 6783 | (2267) | |  | 6483 | (2424) | | |
| Low grade, elementary school | | | | | | |  |  | |  |  | |  |  | | |  |  | | |  |  | | | |
|  | Total step count | |  | 11372 | | (2757) |  | 11333 | (2835) |  | 11922 | (2600) |  | 11702 | (2696) | |  | 11639 | (2673) | |  | 11,158 | (2775) | | |
|  |  | In-school |  | 3960 | | (1435) |  | 4002 | (1585) |  | 4489 | (1407) |  | 3826 | (1405) | |  | 4296 | (1255) | |  | 3902 | (1434) | | |
|  |  | Out-of-school | | | 7412 | (2304) |  | 7331 | (2366) |  | 7432 | (2053) |  | 7877 | (2378) | |  | 7343 | (2094) | |  | 7256 | (2298) | | |
| High grade, elementary school | | | | | | |  |  | |  |  | |  |  | | |  |  | | |  |  | | | |
|  | Total step count | |  | 10313 | | (2622) |  | 10157 | (2723) |  | 10897 | (2537) |  | 10939 | (2655) | |  | 10288 | (2255) | |  | 10007 | (2605) | | |
|  |  | In-school |  | 3758 | | (1348) |  | 3635 | (1249) |  | 3854 | (1371) |  | 3999 | (1377) | |  | 3826 | (1415) | |  | 3653 | (1325) | | |
|  |  | Out-of-school | | | 6554 | (2155) |  | 6522 | (2359) |  | 7042 | (1962) |  | 6941 | (2224) | |  | 6463 | (1687) | |  | 6354 | (2152) | | |
| Junior high school | | |  |  | |  |  |  |  |  |  |  |  |  |  | |  |  |  | |  |  |  | | |
|  | Total step count | |  | 8655 | | (3218) |  | 8064 | (3353) |  | 10232 | (3196) |  | 9351 | (3413) | |  | 8809 | (2941) | |  | 8265 | (3036) | | |
|  |  | In-school |  | 2716 | | (1550) |  | 3458 | (1957) |  | 3514 | (1345) |  | 2862 | (1872) | |  | 2258 | (901) | |  | 2486 | (1291) | | |
|  |  | Out-of-school |  | 5939 | | (2783) |  | 4606 | (2458) |  | 6718 | (2684) |  | 6489 | (3096) | |  | 6551 | (2792) | |  | 5779 | (2583) | | |
