# Supplementary material for: Lower youth steps/day values observed at both high and low population density areas: a cross-sectional study in metropolitan Tokyo
Source: BMC Public Health. 2018 Sep 20;18:1132. doi: 10.1186/s12889-018-6028-y (PMC6149053; doi:10.1186/s12889-018-6028-y)
Supplement: Supplementary file 2 — Table S2. Frequency and percentage of participants with adequate daily total step count by population density subgroups. (DOCX 15 kb) [file 12889_2018_6028_MOESM2_ESM.docx]

| Table S2. Frequency and percentage of participants with recommended daily total step count by population density subgroups. | | | | | | | | | | | | | |  |  |  |  |
| --- | --- | --- | --- | --- | --- | --- | --- | --- | --- | --- | --- | --- | --- | --- | --- | --- | --- |
|  |  |  |  |  |  |  |  |  |  |  |  |  |  |  |  |  |  |
|  |  |  |  | Lowest | |  | Lower | |  | Middle | |  | Higher | |  | Highest | |
| Daily total step count | | |  |  |  |  |  |  |  |  |  |  |  |  |  |  |  |
|  | Boys | |  |  |  |  |  |  |  |  |  |  |  |  |  |  |  |
|  |  | Low grade, elementary school |  | 117 | /227 |  | 50 | /90 |  | 288 | /440 |  | 147 | /219 |  | 648 | /1242 |
|  |  |  |  | (51.5) | |  | (55.6) | |  | (65.5) | |  | (67.1) | |  | (52.2) | |
|  |  | High grade, elementary school |  | 113 | /232 |  | 98 | /146 |  | 321 | /517 |  | 132 | /231 |  | 562 | /1229 |
|  |  |  |  | (48.7) | |  | (67.1) | |  | (62.1) | |  | (57.1) | |  | (45.7) | |
|  |  | Junior high school |  | 113 | /260 |  | 69 | /120 |  | 220 | /467 |  | 97 | /234 |  | 502 | /1246 |
|  |  |  |  | (43.5) | |  | (57.5) | |  | (47.1) | |  | (41.5) | |  | (40.3) | |
|  | Girls | |  |  |  |  |  |  |  |  |  |  |  |  |  |  |  |
|  |  | Low grade, elementary school |  | 116 | /208 |  | 67 | /109 |  | 280 | /481 |  | 125 | /210 |  | 624 | /1245 |
|  |  |  |  | (55.8) | |  | (61.5) | |  | (58.2) | |  | (59.5) | |  | (50.1) | |
|  |  | High grade, elementary school |  | 71 | /215 |  | 60 | /130 |  | 234 | /526 |  | 77 | /214 |  | 397 | /1200 |
|  |  |  |  | (33.0) | |  | (46.2) | |  | (44.5) | |  | (36.0) | |  | (33.1) | |
|  |  | Junior high school |  | 60 | /247 |  | 61 | /125 |  | 187 | /522 |  | 55 | /211 |  | 260 | /1145 |
|  |  |  |  | (24.3) | |  | (48.8) | |  | (35.8) | |  | (26.1) | |  | (22.7) | |
| Percentages were presented as (%). | | |  |  |  |  |  |  |  |  |  |  |  |  |  |  |  |
